# Transformation of Right-Wing Populism in Italy in 2018−2022: From Sovereignism to Patriotism

**DOI:** 10.1134/S1019331622130123

**Published:** 2022-12-23

**Authors:** E. S. Alekseenkova

**Affiliations:** Center for Italian Studies, Institute of Europe, Russian Academy of Sciences, Moscow, Russia

**Keywords:** Italy, right-wing populism, discourse, Brothers of Italy, the League, Forward Italy, elections

## Abstract

The transformation of the discourse of right-wing populist parties in Italy from 2018 to 2022 is considered. Based on analysis of the discourse of the programs and electoral rhetoric of the parties the Brothers of Italy, the League, and Forward Italy, the author concludes that Italian right-wing populism is becoming more moderate, replacing the concepts of *sovereignism* with “patriotism and conservatism” and rejecting Euroscepticism. Although nationalism is still characteristic of the right-wing parties in Italy, it is changing: a legalist approach to migrants is gradually replacing the ethnocultural and socioeconomic approaches. Populism remains the basic strategy of the right-wing parties, but the role of the main “enemy” of the Italian people is shifting from the European bureaucracy to the domestic mainstream Center–Left (i.e., the Democratic Party). The authoritarianism of the right-wing populists has undergone the least change in terms of ideological content between 2018 and 2022, but its importance has increased with the growing public demand for political stability and the increasing personalization of politics. This article contributes to the study of the phenomenon of right-wing populism.

Domestic and foreign scientific studies used to pay great attention to the phenomenon of the emergence of Italian populism; the key features of populist parties and their typologies were considered in terms of the Right and Left ideological spectra and tactical or strategic orientation [Barabanov and Shibkova, 2015; Shibkova, 2019; Maslova, 2017; Zonova, 2019; Piccolino et al., 2022; Emanuele et al., 2021]. However, in general, in both the Russian and foreign literature, discussion continues about the key characteristics and typology of Italian right-wing parties, such as the Brothers of Italy, the League, and Forward Italy: To what extent can they be considered populist, radical, or center–right, what is the relationship between populism and “Euroscepticism” and “sovereignism”? Moreover, the early parliamentary elections in Italy on September 25, 2022, which ended with the victory of a coalition of right-wing parties (Brothers of Italy, the League, Forward Italy, and the Moderates), has provided an opportunity to raise the question of whether Italian populism has undergone a transformation and whether its ideological foundations or methods of political struggle have changed significantly enough compared to the previous elections in 2018? If such changes have taken place, what is their essence, and what direction in the evolution of Italian populism can they indicate?

C. Mudde [Mudde, 2010] pointed to nationalism, populism as a strategy (populism per se), and authoritarianism as three key components of right-wing populism. As part of this work, we will trace the transformation of these three aspects of right-wing populism in Italy in 2018−2022. Critical discourse analysis will be used as a methodology for our research [Fairclough, 2010, p. 132]. In this case, we will focus on the analysis of key concepts and their relationships that form the basis of nationalism, the strategy of populism, and authoritarianism based on policy documents^1^ and speeches and interviews of the leaders of the three key political parties in Italy (League, Brothers of Italy, Forward Italy) in 2018−2022.

The key hypothesis of this study is that right-wing populism in Italy is transforming from a rather radical form to a more moderate stand. This hypothesis is based on three sub-hypotheses: (1) although nationalism is still characteristic of the right-wing parties in Italy, it has changed towards greater inclusiveness: a legalist approach to migrants is gradually replacing ethnocultural and socioeconomic approaches; (2) populism remains the basic strategy of right-wing parties, but, compared to 2018, it relies less and less on Euroscepticism and sovereignism; and (3) the authoritarianism of the Right has undergone the smallest changes in terms of ideological content in the period from 2018 to 2022, but its importance has increased with the growing public demand for political stability and an increase in the personalization of politics.

## RIGHT-WING NATIONALISM: LEGALISM INSTEAD OF NATIVISM?

Nationalism, understood as the ideological mobilization of ethnic or national identity for political purposes, is an integral feature of right-wing populism, along with strategic populism proper (populism per se) and authoritarianism [Mudde, 2010].

A prerequisite for the process of operationalization of nationalism is the “creation” of a nation—outlining the circle of individuals who, according to certain criteria, are included in the nation or, on the contrary, excluded from it. This process of “demarcation of boundaries” between “us” and “them” is of decisive character for right-wing populism: without it, any discursive strategy of populism is a priori unrealizable since it is not clear on whose behalf to conduct a dialogue and build the very opposition between the “people” and the “elites” on whose perception the narrative is aimed, and whose interests are labeled as “national interest.” As rightly noted by P.V. Oskolkov [Oskolkov, 2019], right-wing populism is always a combination of anti-elitism with nativism (nationa-lism).

Setting the goal of delineating the boundaries of the nation, right-wing populism is always exclusive [Vainshtein, 2017]; i.e., it provides for a clear designation of those groups that cannot become part of the “people.” Right-wing populists in different countries approach this task in different ways, using the principle of nativism (the fact of birth) as a criterion of exclusivity, or, for example, the ability and willingness of newly arrived immigrants to integrate into society.

In the camp of the Italian right-wing parties, significant changes occurred by 2022 in the understanding of “us” and “them.” If we compare the election programs of the Brothers of Italy in 2019 and 2022, we can clearly see that the “other” has changed: in 2019, it was the ethnocultural immigrant claiming the social benefits of the state. That is why the principle of *prima gli italiani* was introduced, limiting access to social benefits solely to ethnic Italians. In 2022, under the influence of the Covid-19 pandemic and the Russian−Ukrainian conflict, which has led to a new wave of refugees in the EU, the ethnocultural factor is gradually losing its significance, while the socioeconomic factor remains: the right-wing parties are ready to accept the “other” if he is a refugee from war, while economic migrants looking for a better life remain unacceptable. However, the categories of migrants the shortage of which was obvious during the pandemic can be admitted; in particular, the Brothers of Italy have proposed “green corridors” for agricultural workers. The Islamic factor is practically no longer mentioned in policy documents, and the securitization of Islam is noted to a lesser extent. For example, in 2019, the program of Brothers of Italy declared war on “Islamization”: the construction of places of worship and media and cultural activities funded by “fundamentalist countries” were prohibited; the fight against “integralist proselytism,” which allegedly contributed to the spread terrorism, was proclaimed. The same program said *no* to Turkey’s accession to the EU, proposed limiting the number of foreign students in the classroom, and included an integration policy to avoid the formation of ghettos. The 2022 program only briefly declares that Europe is the birthplace of Judeo−Christian values and declares the fight against “all forms of anti-Semitism, racism, and Islamic integralism.” In fact, only the legalist approach remained at the heart of “nation-building” by 2022: a migrant can be ethnically anyone, but he is accepted if he entered legally. While in 2019 it was stated that immigration is possible according to quotas and only for “those nationalities whose representatives demonstrate a readiness to integrate and do not pose a threat in the field of security and terrorism,” in 2022 the division of nationalities into ready and not ready to integrate no longer exists. The measures proposed by the Brothers of Italy in 2022 are aimed precisely at promoting the legalization of the migration flows, namely, strict joint control of EU borders, the arrangement of primary reception centers for immigrants (so-called hotspots) in the countries of origin to consider issuing refugee status already there, and signing agreements with countries of origin on the prevention of illegal emigration and repatriation. Thus, only illegal immigrants become “others” in 2022. The principle of *primi gli italiani* has disappeared from policy documents. However, at the same time, *ius soli* and all its possible versions are denied as facilitating the possibility of obtaining Italian citizenship.

The League retained a tougher and more detailed approach to immigrants in its program. In particular, the 2022 document proposes the return of the infamous “Salvini decrees,” a sea blockade of ports for vessels of human rights organizations, the introduction of so-called “codes” regulating the actions of NGOs, and the deportation of migrants who have committed crimes. The League proposes to “take in those fleeing war, repatriate economic migrants, ensure real integration for those who receive refugee status, and prevent Italy from remaining Europe’s main refugee camp.” M. Salvini also says *no* to *ius soli* and argues that citizenship should be the result of successful integration, not a condition for it, requiring migrants to “respect the rules and values, culture, and principles of the Western world.” The League insists that immigration must be “skilled” and clearly meet the needs of the labor market. Thus, of all the Italian Rightists, Salvini retains the most rigid approach to immigration issues, demonstrating the most “protective” approach to the concept of citizenship and nation.

Important in the program provisions of the right-wing parties is still a protective approach to the questions of the value and cultural identity of the Italian people. Declaring allegiance to the Judeo−Christian tradition, supporting the traditional family, banning LGBT propaganda, supporting fertility, and fighting the emigration of young people are measures targeted at preventing the negative impact of globalization processes on the value-based and cultural heritage of the country and at preventing the erosion of national identity.

## TRANSFORMING THE POPULIST STRATEGY: REDEFINING THE ENEMY

The second element of right-wing populism, as Mudde defines it, is populism proper as a strategy, i.e., the definition of the “people’s will” in opposition to the will and interests of the “elites,” and articulation of the conflict. The discursive approach to populism considers this element to be key [Laclau, 2005]. The main concepts here are the concepts of *national interest*, which is the “will of the people,” and *sovereignty* as the ability to defend these very “national interests,” actualizing the will of the people. Let us see how the relationships between these concepts were built into the policy documents of the Brothers of Italy, the League, and Forward Italy in 2018−2022.

In 2018, the Brothers of Italy, in fact, did not have a full-fledged program—instead, there were only 12 brief slogans. However, among them there were two clearly defining “the enemies”—the institutions of the EU: one was about “defending national sovereignty from European technocrats,” and the second about protecting the national labor, production, agriculture, and “Made in Italy” products from unfair EU directives. Then, in 2019, the word *sovereignists* (*sovranisti*) appeared on the party’s logo, offering a more extended version of the argument in favor of protecting national sovereignty. The EU is criticized as “a supranational entity ruled by unelected bureaucrats and technocrats, who impose their decisions from above on the peoples of Europe.” Instead, a “European Confederation of Free and Sovereign Nation States” and the restoration of the priority of the Constitution and national legislation over the communitarian law are proposed. The program also claimed that Europe had become an “amusement park” for Germany and France, which used EU institutions in their own interests, to the detriment of the interests of other states and, in particular, Italy. The Brothers of Italy called for intolerance to interference in the internal affairs of the country and “hostile actions” against its national interests. The EU and Germany, in particular, were accused of imposing austerity policies; the EU institutions were accused of acting in the interests of international TNCs and their lobbies, which resulted in a labor market crisis in Italy, the ruin of small and medium-sized enterprises, and the transfer of production to third countries. The single, euro currency, again beneficial only to the Germans, was declared another cause of national troubles. The protection of “Made in Italy” was called “a priority national interest,” as well as the support of fertility and the family, which, from the point of view of the Brothers of Italy, did not receive sufficient attention at the EU level. In order to strengthen the position of Italy in the international arena, it was proposed to introduce popular presidential elections.

However, in the party program for 2022, the “sovranist” rhetoric turned out to be almost completely emasculated. Even the party logo has changed: the signature “Sovranists and Conservatives” has been replaced with “Patriots and Conservatives.” Note that the word *patriotism* was absent in the programs of the parties in 2018 and 2019. In terms of EU reform, the 2022 agenda provides only a modest “restart of the European integration system in favor of a Europe of the fatherlands, based on the interests of the peoples and able to cope with modern challenges.” Italy is invited to become again a “protagonist in Europe and the world” (an idea that has been heard more than once since the mid-1990s), and this proposal took the last, 25th point of the program of the Brothers of Italy. National interest in 2022 is defined in the program of the Brothers of Italy as “protecting the interests of the industrial and production system of the country,” and to this end, Italy should play a more active role in the EU discussion of the Fit for 55 package.

Thus, EU institutions, the single currency, and the norms of the European Union are no longer seen as contrary to the national interests of the country, and the program does not suggest any methods to counteract them. It retains a high degree of protectionism characteristic of the Right and an emphasis on national competitiveness and attaches great attention to conservative values and left-wing socioeconomic ideas, but the confrontation with the EU is practically leveled, thereby reducing the degree of radicalism of the proposed policy. Instead of EU institutions, the anger of the Brothers of Italy in the 2022 program is transferred to the “internal enemy,” namely, to the Center–Left, who have been in power for more than ten years and have driven the country into a severe economic crisis. That is why the program provisions and electoral rhetoric of the Brothers of Italy positioned the elections of 2022 as a milestone, a turning point, which should mark the end of the era of technical governments and finally allow the formation of a “political government” based on the legitimacy of the popular vote. The concept of *sovereignty* is mentioned in the program only once in the form of a quote from the Constitution—“Sovereignty belongs to the people”—and precisely in connection with the illegitimate stay of the Center–Left in power, which was the result not of a popular will but of “behind the scenes games” (*giochi di palazzo*).

The anti-European discourse of the League has also undergone a significant transformation between 2018 and 2022. Thus, in particular, in the 2018 program, in addition to the thesis about the illegitimacy of the EU institutions, consisting of bureaucrats and speculators, there was a call for the EU to return to the state that preceded the conclusion of the Maastricht agreements. It was argued that Italy would be ready to remain in the community only after the revision of all the fundamental documents of the European Union. The euro was declared the main cause of the country’s economic decline. The primacy of national law and the return of national sovereignty were assumed in the following areas: monetary and macroeconomic, and legislative, and in matters of border protection (including the abolition of the Schengen and Dublin agreements). The preservation of sovereignty was declared a national interest, which was to be followed by the country’s foreign policy, which assumed an independent policy in Libya, openness to cooperation with Russia, and maintaining a privileged partnership with the administration of D. Trump, whose fight against Islamic extremism and “aggressive trade and political penetration of China” fully met the national interests of Italy.

The 2022 League program recognizes that Italy’s sovereignty is being eroded by supranational institutions, primarily the EU. However, as a fight against this phenomenon, there is no question of any revision of the EU treaties; instead, it is proposed only to “strengthen the presence” of the Italian leadership in Brussels (in particular, to restore the Ministry of EU Affairs, abolished in 1987) and to return “to the center of the European Union the principle of subsidiarity, which Europe has neglected in favor of solutions imposed at the supranational level to the detriment of states, bringing political decisions closer to citizens disillusioned with an increasingly bureaucratic and distant Europe.” Thus, in the League’s opinion, the principle of subsidiarity and increased representation should help solve the problem of erosion of national sovereignty in the EU. Through negotiations in Brussels, Italy should achieve a review of those EU decisions that, according to the League, are detrimental to the national interests of the country, including the European Green Deal, Fit for 55, and a number of regulatory norms and practices in the field of agriculture. In the same way, it is necessary to influence EU foreign trade policy to promote Italian technologies and products. Similarly, through negotiations, it stipulated the revision of the National Recovery and Resilience Plan, approved in 2021 before the start of the Russian−Ukrainian conflict, which entailed the most severe economic consequences for Italy (which the Brothers of Italy also insist on). Another important element in protecting national sovereignty, according to the League, should be the preservation of the principle of consensus decision making by the EU Council, the destruction of which will become a real opportunity to exclude individual countries from the process of making the most important EU foreign policy decisions and turn the European Union into a form of oligarchy.

As for national interests from the point of view of foreign policy, the program of the League in 2022 does not come into conflict with the EU priorities either. The failures in the implementation of Italy’s foreign policy goals in the Mediterranean are blamed on the Center–Left and not on external forces in the EU: it was the Left that “sought to Europeanize foreign decisions with huge and obvious damage to Italy, which, on the contrary, is interested in creating a network of bilateral agreements with the economies of the Mediterranean countries.” Like the Brothers of Italy, the League calls on the country to become a protagonist on the international stage. The idea of Italy as a mediator of international conflicts and as a representative of NATO and the EU in interaction with third countries is traditionally characteristic of the foreign policy of Rome.

Thus, it is obvious that the Euroscepticism of the League has not completely disappeared but has significantly transformed towards a more moderate view—primarily in terms of methods for strengthening national sovereignty, the main of which is “negotiations” within the EU.

Significant changes along the Euroscepticism−Eurooptimism axis were also introduced into the program provisions of the Forward Italy party. S. Berlusconi’s 2018 program said *no* to austerity, excessive regulation, and bureaucracy and called, like the League, for the revision of the EU treaties, the redistribution of Italy’s payments to the EU budget, the priority of national law over EU law (“restoration of sovereignty”), and the protection of “Made in Italy” and agricultural producers. The 2022 agenda is completely different. Even its title, which can be translated literally as “Today as Never Before—the Choice of the Camp” (*Oggi piu che mai una scelta di campo*), underlines the priority of the international agenda and the desire to emphasize the choice of Italy—together with the EU and NATO—in the confrontation between Russia and the so-called “collective West.” The very first lines speak of adherence to the liberal, Christian, pro-European tradition and values and principles of Western civilization. There is also the main slogan, “Italy is fully part of Europe, the Atlantic Alliance, and the West. More Italy in Europe, more Europe in the world,” which became the first point of the common program of the center-right coalition in the 2022 elections. There is not a single mention of “sovereignty” or “national interests” in the program. The section on foreign policy and defense is called “We are Atlanticists and Europeanists.” It proposes the promotion of a common foreign policy of the EU, a transition from a consensus vote to a qualified majority in the European Council (where Forward Italy is fundamentally at odds with the League), the creation of a European army, a revision of the Stability Pact, the mandatory distribution of immigrants by quotas within the EU, support for NATO, strengthening relations with the United States, the European “Marshall Plan” for Africa, etc.

Nevertheless, it is worth noting that, even in 2018−2019, Berlusconi’s attitude to the phenomenon of sovereignism was very ambivalent. For example, in a 2019 interview, he argued,

Sovereignty is a deception that needs to be abandoned, it is a stupid idea, and those who believe in it are stupid. A nationalist and sovereign Europe was the cause of two world wars and tens of millions of deaths. Do we want to return? No. With sovereignty, we will end Le Pen in France, who has many votes but cannot govern.

In 2022, he confirmed his thesis:

It is not difficult for me to repeat this. Our Center–Right have nothing to do with the far-right components that exist in other countries, but in Italy they, fortunately, do not matter, because we have many democratic Rightists. Our presence [in the center-right coalition—*E.A.*], I repeat, is a guarantee of the democratic, pro-European, and Atlantic vocation of the coalition. Otherwise, we would not be there.^2^

The common thesis that the party shares with its coalition partners is now only the desire for maximum subsidiarity within the EU:

We want Europe to be a community governed by democratic principles, with the direct election of the president of the EU Commission by European citizens and overcoming the principle of unanimity in the European Council. We want to move towards a model based on subsidiarity, which guarantees maximum unity and at the same time maximum autonomy of individual countries, as is the case in the United States. Finally, we want Europe to join NATO and the West without hesitation.^3^

Thus, we can state that by now Forward Italy has almost completely abandoned Euroscepticism.

The common program of the center–right coalition of 2022 also clearly shows a decrease in the level of Euroscepticism. There is not a single mention of sovereignty in it, and the concept of national interest occurs only once, where the program dwells on the “protection of national interests when discussing European legislative dossiers, including in the light of changes in the international context, with special emphasis on ecological transition.” The common program of the Center–Right begins with words borrowed from Forward Italy: “Italy is fully part of Europe, the Atlantic Alliance, and the West. More Italy in Europe, more Europe in the world.” It also expresses full commitment to the process of European integration with the prospect of becoming a union more political than bureaucratic; the desire to reform the Stability and Growth Pact is expressed. It declares compliance with the obligations assumed in the Atlantic Alliance, including in matters of defense appropriations, support for Ukraine, and any diplomatic initiative aimed at resolving the conflict.

It is also interesting to note that the foreign policy agenda is included in the first paragraph of the common program, which is not observed in the programs of the parties themselves. It is obvious that this step was aimed at “calming” Italy’s European and overseas partners that even with the Center–Right in power, Rome will not change its Euro-Atlantic course. Given the program narrative and pre-election discourse of these same parties on the eve of the 2018 national elections and the 2019 European Parliament elections, Brussels and Washington had every reason to worry about how Italy would behave in the most difficult times of an armed conflict in the heart of Europe and tough confrontation with Russia. However, Russia’s military operation in Ukraine became a factor that radically changed the position not only of official Rome but also of right-wing populists, both in relation to Moscow and in relation to Western partners. Russia’s actions were perceived as aggression and an unjustified violation of international law, and unity with the West and the EU was a response that actually crossed out all previous years of confrontation with Brussels and the European bureaucracy. Therefore, on the eve of the 2022 elections, the Center–Right did everything to convince international partners of loyalty to Italy’s traditional alliances. On September 22, 2022, G. Meloni declared “support for Ukraine without any hesitation or doubts.” She also said, “We believe that Italy’s national interest today is not to appear as a weak link of the West but to respect fully our international alliances.”^4^*Thus, while in 2018 the national interest indicated in the programs of the Right was the revision of all EU treaties, today it has been transformed into the task of “not appearing as a weak link of the West.”*

The persistence of the conflict between the “people” and the “elites” in the discourses of center–right parties indicates that they are still committed to the populist strategy (populism per se)—with the only difference being that, compared to 2018, the European bureaucracy has ceased to be their main target: the role of the main “enemy” of the Italian people has actually been completely transferred to the Center–Left, in particular, to the Democratic Party, with which the work of “technical governments” and all the unpopular economic measures implemented by them in previous years were inextricably linked. It seems that the main reasons for this transformation were (1) Italy’s dependence on EU financial assistance in the implementation of the economic recovery plan and (2) the Russian−Ukrainian conflict, which contributed to the growth of pan-European solidarity.

## IS RIGHT-WING POPULISM AUTHORITARIAN IN ITALY?

There is no unambiguous opinion in the scientific literature about the extent to which right-wing populism is authoritarian, nor about whether this characteristic refers to the populist project of the future society rather than to the populist parties themselves and the style of leadership. For example, Mudde calls authoritarianism one of the three key elements of right-wing populism, referring to their ideas about a tightly controlled society. E. Laclau [Laclau, 2005] speaks of the inevitability of authoritarianism due to a populist leader’s function of the aggregator and articulator of public opinions and sentiments. Other researchers [Baranov, 2015] speak of a special psychotype of the populist electorate, which determines their choice in favor of a strong leader. The authoritarian component of populism is especially emphasized by the concept of P. Norris and R. Inglehart [Norris, Inglehart, 2019], emphasizing that right-wing populists advocate tight control in the security sector, display xenophobia, call for control over moral values, etc.

If we consider from this point of view Italian right-wing populism in dynamics from 2018 to 2022, we will see that all the above elements of authoritarianism are somehow inherent in it and remain relevant, but the balance between them changes depending on public sentiments and expectations, as well as the nature of domestic political and external challenges.

Perhaps the most stable characteristic of these parties is that all of them—the League, the Brothers of Italy, and Forward Italy—are parties of the leader type, headed by leaders with fairly great charisma. Moreover, over the past five years, the degree of personalization of power within the parties has increased. All three parties practice direct communication with voters; actively use social networks, new technologies, and the so-called “square diplomacy”; constantly exploit the image of “a person of the people”; etc.

Also quite stable is the presence of security-related issues in both program provisions and the rhetoric of the right-wing parties. These issues range from problems caused by illegal immigration to bills aimed at protecting housing, the right to self-defense, reforming law enforcement and the penitentiary system, etc. That is why Salvini became Head of the Ministry of Internal Affairs in 2018 and sought to do it again, after the elections in 2022. The same range of issues includes the securitization of immigration and religion, in particular Islam, which we spoke about above. All these elements remain relevant for the right-wing parties in 2022.

The presence of proposals for reforming the Constitution of the country towards presidentialismin in the programs of the right-wing parties also persists. Many internal and external opponents of the Italian right populist see this as an authoritarian threat. Indeed, in both 2018 and 2022, one of the key pillars of the center–right agenda was institutional reform. The society’s demand for a more stable political system without annual political crises; the desire to gain more “responsible” political leadership; and the general trend towards the personalization of power, which was entrenched during the pandemic [Alekseenkova, 2020], have become important factors in Italian politics. The level of trust in political and administrative institutions in Italy has not exceeded 20−30% for more than a decade [Ladini, 2021]. At the same time, the level of personal trust in many “professionals” who came to the government during the pandemic, including Prime Minister M. Draghi himself, turned out to be very high (more than 65%).

In response to this demand for the personalization of power, the Rightists propose the transformation of the political system towards presidentialism: the introduction of universal direct presidential elections and the electability of the prime minister. According to Meloni, the lack of political stability is the main source of the country’s economic problems. The League’s program develops the populist thesis about the loss of the people’s influence on decisions, which are often the result of collusions between parties or a political situation. Direct presidential elections should increase responsibility for the decisions made and thus contribute to the growth of trust. As a successfully working model, Salvini proposes to use the experience of the elected heads of regions and communes of Italy. A popularly elected president must also unite the nation, which still has many rifts within it. Indeed, public opinion polls show that more than 80% of supporters of the Center–Right and more than 60% of the electorate of the Democratic Party would welcome the introduction of direct popular elections for the president of the country.^5^

However, in the view of the Italian Rightists themselves, presidentialism does not mean at all a movement towards authoritarianism, but, above all, an increase in the responsibility of the leader. In addition, the risk of authoritarianism, from their point of view, should be offset by the consolidation of real regional autonomy—maximum horizontal subsidiarity, which fully meets the task of “bringing power to the people,” direct democracy, and citizens’ participation in government.

Regional autonomy, introduced in the 1970s and continued through the constitutional reform of 2001, has not been fully implemented thus far. From 2001 to 2017, in a number of regions of the country, referendums were held on granting greater autonomy (Lombardy, Veneto); however, this process has never been completed. The League’s program assumes “federal regionalism” based on the principle of differentiated autonomy of the regions. The idea of greater autonomy still appeals to those regions of Italy (mostly northern) that believe that they can be more successful if subsidiarity is maximized and central intervention is minimized. The success of some of them in the fight against the pandemic has further strengthened their belief.

As in any study of populism, it is rather difficult to say to what extent the authoritarianism of the Right was a response to the public demand for greater stability and accountability of power, and to what extent this demand was formed under the influence of the populist discourse about the need for institutional reforms. Nevertheless, for several years now, researchers have noted the fatigue of Italian society from the permanent governmental leapfrog and, as a response to this, the demand for stability, the personalization of power, and its accessibility for ordinary citizens. However, dissatisfaction with the “emergency” management style that was formed in Italy under the influence of the global economic crisis of 2008−2011, and then the Covid-19 pandemic, and the energy crisis of 2022 is also recorded as a countertrend. Note that criticism of this second trend also comes from the same right-wing populists^6^ who accuse the Prime Minister of usurping power and removing parliament from the process of making key decisions.

Given the above, it is certainly appropriate to talk about the presence of authoritarian elements in the discourse of right-wing populists, as well as about the corresponding demand of society, but there is also a countertendency and it is deeply rooted in Italian political culture that rejects any attempt to reduce pluralism and any unification, be it either local or regional.

## CONCLUSIONS

The documented transformation of right-wing populist discourse, indicating a rejection of hard forms of nationalism, Euroscepticism, and sovereignism, seems to have allowed the Center–Right to increase the level of electoral support. Right-wing populism in Italy is becoming more moderate. The emasculation of the word *sovereignty* and the increasingly frequent replacement of it with the terms *patriotism* and *national interests*; the transfer of “blame” for Italy’s economic and geopolitical difficulties from Brussels to the Center–Left; the cancellation of calls for the revision of European treatises; and even a more moderate, “legalist,” narrative in relation to migrants, combined with the promotion of traditional values, allowed the Right to win the support of 44% of Italian citizens, despite the rather harsh rhetoric of the Center–Left, trying to present the Right as the heirs of fascism and the main supporters of Putin and Orbán in the EU. It is also worth noting the strengthening of the “left” component in the discourse of the right-wing parties in the form of economic measures to support families and enterprises and protectionism for “Made in Italy”—a trend that many researchers spoke about back in 2017 [Global right-wing rebellion, 2017] and that is gaining momentum due to the deterioration of the international economic situation against the backdrop of the Russian−Ukrainian conflict. “Losers from globalization” [Baranov, 2015, p. 26; Pogorel’skaya, 2004] in today’s situation turn out to be “losers from deglobalization,” which has intensified under the influence of the Russian−Ukrainian crisis.

Seeing the sole lifeline in the EU and NATO, right-wing populism in Italy has directed all criticism towards the “internal enemy.” Having completely abandoned “nonsystemic” initiatives in the form of withdrawing from the EU treaties or the euro area, it is increasingly drifting towards “corrective to democracy” [Rovira Kaltwasser, 2012]. By reducing the degree of radicalism, right-wing populists are building up support for the electorate that is not ready to face a new global crisis. However, it seems premature to talk about whether this transformation is tactical or strategic, and whether the remaining contradictions between Italy and the EU will once again become a reason for raising the discourse on national sovereignty in the event of a further deterioration in the international economic environment.

## Footnotes

1 Il programma di Fratelli d’Italia, 2018. https://www.fditrieste.it/2018/01/schema-programma-politiche-2018/; Il programma di Fratelli d’Italia, 2019 (elezioni europee). chrome-extension://efaidnbmnnnibpcajpcglclefindmkaj/https://www.fratelli-italia.it/wp-content/uploads/2019/04/Programma-completo-1.pdf; Il programma di Fratelli d’Italia 2022. https://www.fratelli-italia.it/programma/; Programma di governo Lega 2018. https://www.leganord.org/component/phocadownload/category/5-elezioni?download=1514:programma-lega-salvini-premier-2018; Lega. Programma di governo 2022. https://legaonline.it/; Per l’Italia. Accordo quadro di Programma per il governo di centrodestra 2022. chrome-extension://efaidnbmnnnibpcajpcglclefindmkaj/http://www.forzaitalia.it/speciali/PER_L_ITALIA_ Accordo_quadro_di_programma_per_un_Governo_di_centrodestra.pdf; Programma elettorale Forza Italia 2018. chrome-extension://efaidnbmnnnibpcajpcglclefindmkaj/http://www.forza-italia.it/speciali/Programma_centrodestra_condiviso_10_PUNTI.pdf; Programma Forza Italia 2022. https://www.corriere.it/elezioni/22_settembre_25/programma-forza-italia-fd4d201a-3b5a-11ed-8e93-4aa9ade4f3e7.shtml.

2 L’Europa cammina con noi. Intervista al Cav: “Il sovranismo è un’idea stupida,” *Il Foglio*, Aug. 19 (2022). https://www.ilfoglio.it/politica/2022/08/19/news/l-europa-cammina-con-noi-intervista-al-cav-il-sovranismo-e-un-idea-stupida–4339987/.

3 Ibid.

4 Meloni: “Siamo al fianco degli alleati, niente crepe nell’Occidente,” Agi.it, Sep. 22 (2022). https://www.agi.it/politica/ news/2022-09-22/ucraina-meloni-a-fianco-alleati-no-italia-anello-debole-18174942/. Cited September 22, 2022.

5 Sablone, L. (2021) Gli italiani vogliono il presidenzialismo, *Il Giornale*, Dec. 13 (2021). https://www.ilgiornale.it/news/politica/sondaggio-sul-presidenzialismo-lelezione-diretta-piace-74-1995578.html.

6 La lettera di Salvini che accusa il governo sull’emergenza, Today.it, Sep. 6 (2020). https://www.today.it/politica/lettera-salvini.html (cited October 10, 2022).
